# Caregiver Experiences of a Peer Mentor Family Physical Activity Programme in England: A Qualitative Interview Study

**DOI:** 10.1111/cch.70053

**Published:** 2025-02-24

**Authors:** Rebecca Symes, Leah Jayes, Elizabeth Orton

**Affiliations:** ^1^ Lifespan and Population Health, School of Medicine The University of Nottingham Nottingham UK; ^2^ Leicestershire County Council Leicester UK; ^3^ Public Health Specialty Training Programme East Midlands UK; ^4^ Institute of Health and Allied Professions Nottingham Trent University, Clifton Campus Nottingham UK

**Keywords:** barriers, behaviour change, children, family, peer mentor, physical activity, wellbeing

## Abstract

**Background:**

Physical inactivity is a major risk factor for developing chronic disease and contributes to health inequalities. Many children and adults do not achieve recommended physical activity targets. Active Families was a pilot programme that aimed to increase physical activity in families in the East Midlands, UK, using volunteer peer mentor support. This study aimed to explore caregiver experiences of family physical activity in participants of the programme Active Families.

**Methods:**

Qualitative, semi‐structured interviews were conducted with 13 caregiver participants of Active Families. Interview transcripts were explored using thematic analysis.

**Results:**

Most caregivers reported increased family physical activity and improvements in health and relationships. Attitudes towards family physical activity became more positive and role modelling encouraged families to be active. Volunteer peer mentors aided families using behaviour change techniques and provided psychological, emotional and practical support. Volunteers maintained programme delivery during the coronavirus pandemic, and exercise was used by some as a coping strategy. Some older children did not engage well with the programme, and maintaining physical activity was a challenge for others.

**Conclusion:**

Caregivers reported improved experiences of family physical activity, with positive impacts on wellbeing and family life reported. The family–volunteer relationship appeared to be key. Behaviour change techniques and providing holistic support should therefore be considered when designing family physical activity programmes. Further research is needed to understand how best to engage older children in family physical activity and ensuring physical activity is maintained.

**Summary**

- This study presents a rich account of caregiver experiences and provides an understanding of likely mechanisms behind successful family physical activity behaviour change.
- Caregivers reported improved experiences of family physical activity, including positive impacts on wellbeing and family life.
- The family–volunteer relationship appears key to success. Volunteer peer mentors used behaviour change techniques and provided psychological, emotional and practical support.
- The holistic, tailored support provided by peer mentor volunteers and the use of behavioural change techniques should be considered when designing family physical activity programmes.

## 1 Introduction

Physical inactivity is one of the main risk factors for developing chronic disease (Nielsen 2020). Physical activity (PA) in children and young people is associated with improved cardiovascular fitness and educational attainment (Norris et al. 2020; Centers for Disease Control and Prevention 2022), and in adults, it has been shown to be protective for chronic conditions and mental health problems (Public Health England 2016). There are wider benefits of PA in adults and children including building stronger communities through participation in PA and lower health and social care system costs due to improved health and greater independence (Public Health England 2016).

However, in the United Kingdom, only 63% of adults (Sport England 2023) and 47% of children and young people (Sport England 2022) in 2021/2022 were meeting the UK Chief Medical Officer guidelines of 150 min per week for adults and 60 min a day for children of PA. People living in the least affluent areas are twice as likely to be physically inactive compared to those in the most affluent areas (Public Health England 2016; Drenowatz et al. 2010). More recently, the coronavirus pandemic has also negatively impacted PA levels (Sport England 2021). Interventions to increase PA are therefore needed.

There is some uncertainty surrounding the impact and mechanisms of success for family PA interventions. Studies in the United States have found higher levels of PA in children who have parents engaged in PA (Cleland et al. 2011). However, randomised control trials of family PA interventions carried out in the United Kingdom and Canada have found no long‐term significant change in child PA levels (Rhodes et al. 2019; Guagliano et al. 2020).

Additionally, several studies worldwide have shown benefits of peer mentor support for PA levels (Dorgo et al. 2009; Cox et al. 2018). Peer mentoring is a form of mentorship that usually takes place between a person who uses their experience to support a person who is new to an experience. Peer mentors have been shown to provide additional support and facilitate increased social interaction during PA interventions in older adults (Crozier et al. 2020). Whether the benefits of peer mentor led programmes extend to family PA interventions requires further study.

Sport England, a non‐departmental public body of the UK government (Sport England 2025b), has invested in pilot projects across England focusing on families in lower socio‐economic groups (Sport England 2025a). One of these projects, the Active Families programme, was based in the East Midlands. It aimed to support families to be active together, engaging families through the development of bespoke PA plans with volunteer peer mentor support. The project hypothesised that if families engage in PA together, they are more likely to motivate each other and maintain PA (Spokes 2021).

The programme was delivered by an established East Midlands charity in collaboration with local government and leisure partners. Families were recruited from lower socio‐economic backgrounds, with at least one child aged 5–15 years who had been referred for additional early help services. Charity coordinators assessed PA needs with families and co‐produced bespoke activity plans. Volunteer peer mentors then provided intensive support by providing weekly visits to families to review activity plans, motivate, and engage in activities such as games or walking with families. Families were initially offered 6 months of engagement with the peer mentor, with the option to extend their enagement to 1 year. The caregivers interviewed started their engagement with the programme between September 2019 and early March 2020. During the development of this programme, there was severe social disruption due to the global coronavirus pandemic, with all caregivers interviewed engaging with the programme during this time. Adaptations were made to deliver the programme remotely by telephone or socially distanced meetings in line with national and local guidelines.

The aim of this qualitative study was to explore caregiver experiences of family PA in those partaking in the Active Families programme. Semi‐structured interviews were used to identify caregiver attitudes towards family PA (including during periods of adversity such as the coronovirus pandemic), assess the family‐perceived value of Active Families volunteer peer mentors and explore the barriers and facilitators to PA.

## 2 Methodology

Semi‐structured interviews were completed with caregivers to gain a deeper understanding of the whole family experience of the Active Families programme. A constructionist approach was taken to inform a rich interpretation of participants' accounts (Gibbs 2007; Clarke and Braun 2017). Qualitative descriptive methodology was used to describe experiences and gain insights from caregiver participants about the Active Families programme (Kim et al. 2017).

### 2.1 Interview Guide

The interview guide was developed collaboratively among the authors (R.S., L.J. and E.O.), Active Families programme coordinators and a review of relevant literature. The authors are public health professionals with qualitative research experience. Three interviews served as pilot interviews and were conducted by R.S. and reviewed by L.J. Minor changes to the interview guide made to improve flow. Interviewees were encouraged to expand on topics pertinent to the study aims.

### 2.2 Study Population and Participant Recruitment

Adult caregivers who were receiving support from the Active Families programme, or had done so in the last 6 months, were purposively sampled. The aim, and criteria used for sampling, was to recruit participants from each of the seven district local authority areas in the catchment region for the programme and families at different stages of programme participation.

The Active Families programme coordinators contacted caregivers to ascertain interest in partaking. The research team then contacted potential participants to provide a participant information sheet and answer any questions. If the caregiver wished to partake, a telephone interview was arranged at their convenience and verbal consent sought prior to participation.

### 2.3 Data Collection

Interviews were completed in English by R.S. and audio‐recorded. A short demographic questionnaire was completed prior to the interview by interview participants. Participants were free to withdraw at any time without affecting their future Active Families support. Participants received a £20 voucher for taking part. The number of participants recruited ensured sufficient information power for this study to develop new knowledge and meet the aims of the study (Malterud et al. 2016).

### 2.4 Data Analysis

Thematic analysis was conducted using six steps outlined by Braun and Clarke (2006) and Terry et al. (2017). R.S. familiarised herself in the data by reading and re‐reading each transcript, and reflections were noted (Vaismoradi et al. 2016). Codes were then generated using an inductive approach to categorise data into clusters of similar meaning (Stuckey 2015). To provide researcher triangulation and enhance validity, four transcripts were independently double‐coded by L.J. (Carter et al. 2014). R.S. and L.J. then met to discuss their initial themes/subthemes and were largely in agreement (Patton 1999). A consensus approach was used if there were disagreements and joint decisions reached (O'Connor and Joffe 2020). The number of transcripts double‐coded was determined by the number needed to form the initial themes/subthemes and when no new themes emerged.

These initial themes/subthemes were revisited and refined throughout the analysis process to ensure they were an accurate reflection of the data and were reviewed by the research team (Stuckey 2015; Vaismoradi et al. 2016). NVivo 12 was used to support management of the data. The themes and subthemes were written up with associated quotes to provide a vivid description of the participants' accounts (Braun and Clarke 2006; Gibbs 2007).

For the demographic questionnaire data, descriptive statistics were calculated. Index of multiple deprivation (IMD) was obtained from participant postcode provided in the questionnaire. IMD is used to classify relative deprivation as a measure of poverty in a small residential area. Deciles are calculated using a national ranking of IMD. Participant district is the administrative local government area where a caregiver lives. Routinely collected demographic data were extracted from an Active Families service delivery database. This enabled descriptive comparisons of ethnicity and disability status between the wider Active Families cohort and interview participants.

## 3 Results

A total of 16 people expressed an interest in participating and 13 caregivers took part. Table 1 (see Supporting Information) outlines participant characteristics. Routinely collected programme demographic data from the wider Active Families programme showed the majority (88%) of participants were of White British ethnicity. A third (32%) of participants were recorded as having a physical or mental health condition. This was compared to the participants in this study where 100% were of White British ethnicity and 30.8% of participants reported a disability.

**TABLE 1:** Participant characteristics.

	All participants (*n* = 13)
Participant sex	
Female, *n* (%)	12 (92%)
Participant age (years)	
Mean	43.4
Range	32–57
Ethnic background	
White British, *n* (%)	13 (100%)
Participant district (administrative local government area)	
A, *n* (%)	5 (38.5%)
B, *n* (%)	2 (15.4%)
C, *n* (%)	2 (15.4%)
D, *n* (%)	1 (7.7%)
E, *n* (%)	2 (15.4%)
F, *n* (%)	1 (7.7%)
Index of multiple deprivation postcode deciles for participant household (1 = *most deprived*, 10 = *least deprived*)	
1–2, *n* (%)	0 (0%)
3–4, *n* (%)	2 (15.4%)
5–6, *n* (%)	4 (30.8%)
7–8, *n* (%)	6 (46.2%)
9–10, *n* (%)	1 (7.7%)
Employment status of participant	
Paid employment, *n* (%)	4 (30.8%)
Not employed, *n* (%)	4 (30.8%)
Unemployed, *n* (%)	4 (30.8%)
Did not answer, *n* (%)	1 (7.7%)
Participant caregiver role	
Parent, *n* (%)	12 (92%)
Grandparent, *n* (%)	1 (8%)
Number of adults in the household (18 years and over)	
1	4 (30.8%)
2	9 (69.2%)
Number of children in the household (under 18 years)	
1, *n* (%)	5 (38.5%)
2, *n* (%)	3 (23.1%)
3, *n* (%)	3 (23.1%)
4, *n* (%)	1 (7.7%)
5, *n* (%)	1 (7.7%)
Participants reporting a disability	
Yes, *n* (%)	4 (30.8%)
No, *n* (%)	9 (69.2%)
Participants reporting someone else in their household as having a disability	
Yes—child in household with disability, *n* (%)	6 (46.2%)
Yes—other caregiver in household with disability, *n* (%)	1 (7.7%)
No, *n* (%)	6 (46.2%)

Interviews took place between August and December 2020 and were between 25 and 53 minutes (median, 38 minutes).

Five themes were derived from the dataset and are presented below with supporting quotes. Each supporting quote is described with a participant identifier, sex, age and number of children under 18 in the household (e.g., P001, F, age 27, 2 children).

### 3.1 Theme 1: Before Active Families: PA Levels, Barriers and Motivations for Participating

#### 3.1.1 PA Levels as a Family

Prior to being involved in Active Families many families described being relatively inactive as a family, occasionally swimming or walking, with little motivation to leave the house.

#### 3.1.2 Barriers to Being Active

Child behaviour problems or needs were a barrier for some participants and caregivers demonstrated anxiety towards family PA because of this. One caregiver described feeling ‘guilty’ for not being active with their children to avoid the stress of family activity. Participants knew the benefits of PA and wanted to be active, but this was sometimes outweighed by experiencing child behavioural problems. Mental health problems in caregivers were also described as a barrier: ‘At the time I was struggling with depression and isolation so we weren't actually doing that much. We spent most of our time in the house’ (P002, F, age 33, 1 child).

#### 3.1.3 Motivations for Participation

These barriers and the low levels of activity formed many of the reasons for involvement in Active Families. For some caregivers the primary motivation for participating was to improve their own levels of activity; using personal pronouns such as ‘I’ and ‘me’: ‘My daughter is quite active anyway so I never really worry about her but it was just more for me’ (P006, F, age 32, 1 child). The mental and physical health of caregivers were also reasons for seeking support. Some participants spoke about severe mental health problems and lack of confidence. There appeared to be a belief among most of the caregivers that Active Families would help them manage their current mental health challenges: ‘Because I just felt that I needed to speak to someone. I felt that if I didn't speak to someone, this is going to be, sorry, this is really hard to say, but basically, I was on the verge of just going, disappearing’ (P009, F, age 48, 1 child). For other caregivers their primary motivation was to improve the wellbeing and increase PA of others in their family, many of whom had become overly reliant on technology such as TV. Aiming to improve child behaviours was another motivation to partake. Some participants explained spending more time as a family was a key motivator: ‘There was nothing that we saw as unifying us as a family …’ (P003, M, age 57, 3 children).

### 3.2 Theme 2: Referral and how the Active Families Programme Was Delivered

#### 3.2.1 Referral and Signposting to the Programme

Many of the caregivers were introduced to Active Families by health and social care professionals and were already involved with support services. One caregiver had been introduced to Active Families in an informal way through peers on social media due to experiencing anxiety and isolation.

#### 3.2.2 Level of Family Engagement With the Volunteer

Most caregivers explained the whole family had been involved in Active Families. However, in a couple of families, older children and other caregivers did not participate due to college/work commitments. Nearly all participants discussed weekly contact with their volunteer. When coronavirus restrictions allowed, families went on walks with their volunteer. Many caregivers felt the delivery of the programme had been adversely impacted to some extent by the coronavirus pandemic. Some had not been able to engage with their volunteer in person: ‘It was a bit difficult because when we couldn't be involved with Active Families as much as what we'd like to have been. We still tried to get out during those weeks that we weren't having any contact [with the volunteer] but it wasn't particularly easy’ (P005, F, age 47, 1 child). It was often only the caregiver that was able to talk to the volunteer via the telephone, rather than the whole family: ‘I think just not being able to have the face‐to‐face, not being able to meet the kids and know about, you know, just talk to them [the children] instead of talk about them [the children] through me as well’ (P001, F, age 47, 2 children).

#### 3.2.3 Maintaining PA During the Programme

The volunteers and charity coordinators provided activity ideas and signposted to local sports activities. Some caregivers spoke of financial support with swimming vouchers. A few were provided with equipment such as activity monitors, which helped motivation. Participants indicated how adaptable the volunteers had been to their family's needs: ‘… they went out their way and they got me some leaflets and some pictures … and I thought that was really amazing, because that was one thing I did miss, going to the gym’ (P009, F, age 48, 1 child). Despite the challenges of the coronavirus pandemic, all participants appreciated how volunteers had adapted the programme. Caregivers described being sent activity packs and being in regular contact with the volunteers by telephone. This consistent contact provided support and motivation for maintaining PA to some extent ‘… it's been nice that it's carried on actually, it's been very important to me that it carried on through lockdown because even myself, a very active person, came to doing nothing!’ (P001, F, age 47, 2 children).

### 3.3 Theme 3: The Family–Volunteer Relationship

In addition to practical support, the family–volunteer relationship was described as providing encouragement, emotional support, and building confidence, which resulted in behaviour change.

#### 3.3.1 Accountability and Encouragement

All caregivers spoke extremely positively of the volunteers, suggesting the important impact of the volunteer support. Weekly calls/visits were a motivator. Volunteer encouragement was described as important for changing PA behaviours: ‘So we're both egging each other on because she's trying to get fit as well and lose a little bit of weight and I said, “That's what my main goal is, to get more active and lose weight”’ (P006, F, age 32, 1 child).

#### 3.3.2 Support and Friendship

Overwhelmingly the volunteers were described as providing emotional and psychological support and forming meaningful friendships with the caregivers. Many described this support as the best thing about the programme: ‘Again that was nice, to have [volunteer] on the phone, she was more like a psychologist I suppose some days! That was very helpful for me, I know it's not all activity but it's the wellbeing’ (P001, F, age 47, 2 children). Some caregivers suggested they were socially isolated, and the volunteers provided adult conversation and company. For one mother, her volunteer appeared to fulfil the role of confidante where there was a lack of family support in her social environment. Furthermore, some of the volunteers provided holistic support to their families, including practical help in their homes.

#### 3.3.3 Confidence Building

Caregivers described how the support and empowerment provided by the volunteers improved their parental self‐confidence and had positive impacts on mental wellbeing and activity. The volunteers drew on their own experiences of being a parent and this provided reassurance they are not the only parents to experience challenges: ‘They'd make me feel better about myself like just as a person and as a mum …’ (P004, F, age 37, 4 children).

#### 3.3.4 The Wider Family

Some volunteers were able to connect with the children by discussing common interests. Other caregivers, especially those with teenagers, described little interaction with the volunteer. This was attributed to the children not wishing to engage due to their intrinsic age and personalities: ‘I know my brood would have absolutely rejected anything like a Zoom call or a face‐to‐face, it's hard enough to get a face‐to‐face with their nanny, let alone a stranger’ (P001, F, age 47, 2 children).

### 3.4 Theme 4: Barriers and Facilitators to PA in the Context of Active Families

#### 3.4.1 Barriers to Continued PA

Fear, anxiety and low mood were described as ongoing barriers and were exacerbated by the coronavirus pandemic. Child behaviours also continued to be a challenge: ‘Because [child 4] can be quite out of control, I always feel insecure, inferior, because you always get someone who feels they [the public] need to have their say’ (P011, F, age 38, 5 children). Practical issues inhibited families' abilities to be active. A lack of transport, limited facilities such as leisure centres and the cost of activities contributed to feelings of isolation and a lack of opportunity: ‘When you can't drive, to me, being in one of these towns is like living on a desert island’ (P005, F, age 47, 1 child). Poor weather was viewed as an obstacle. A lack of motivation, particularly due to the psychological and social challenges of the coronavirus pandemic, was given by some as reasons for not continuing with PA after the programme. A lack of time was also suggested as a barrier.

#### 3.4.2 Facilitators to Staying Active

Social support provided by the volunteers was a key motivation for many and this appeared to improve feelings of loneliness: ‘I don't know whether it's just because I need that interaction with another adult so that I don't feel like I'm totally on my own and then she's got someone to play with as well’ (P006, F, age 32, 1 child). For some, the coronavirus pandemic provided an opportunity, with families spending more time together, providing encouragement and improving closeness. Material factors such as vouchers provided by the programme helped to relieve anxieties towards affordability. A few participants found fitness technology motivating. For example, an activity tracker (Fitbit) helped one participant by setting step targets and giving reminders to be active. Seeing the positive results of being active was an important facilitator for some. PA was instrumental for some families to maintain good wellbeing during coronavirus restrictions. Looking to PA after the programme, building habits and goal setting were seen as important. Some caregivers suggested a change in attitudes towards PA, resulting in exercise becoming a routine part of family life: ‘It will just become so entrenched in our DNA that we wouldn't slide into inactivity, we're more likely to go the other way and become more active rather than less and less active as people …’ (P003, M, age 57, 3 children).

### 3.5 Theme 5: Active Families Programme Outcomes

#### 3.5.1 Increased PA as a Family

Many caregivers spoke of having increased opportunities for family PA with and without their volunteer. Sometimes caregivers described activities that would not be typically seen as exercise but led to an increase in activity, such as shopping.

Being active as a family was a key motivation for some. One caregiver described the knock‐on effect of PA within their family, suggesting the motivation that can be drawn from observing siblings exercise. Seeing the involvement of a parent seems to have provided encouragement for children in one family: ‘I think it's really highlighted the fact that because Mum's speaking to somebody every week about activities, the girls, they're all aware so it's made them just aware of it, that just sitting on your bum watching TV and your phones and things, it's just not a happy life!’ (P001, F, age 47, 2 children). Attitudes towards family PA appear to have changed for some caregivers, with more confidence to be active as a family and more openness to try new activities. Most participants were positively impacted by spending more time together as a family. Caregivers described the time they spent together as bonding and improving relationships: ‘Because when you do something together as a family, it goes well, it makes you feel good or better as a family, whereas if you just have to keep doing things individually you never really get a proper bond, things like that’ (P008, F, age 48, 3 children).

#### 3.5.2 Limited Impact on Family PA

A few caregivers also explained there had been no change in PA levels since involvement in the programme. These families tended to have older children that had not engaged well. Some caregivers suggested potential reasons for the lack of family activity, such as difficult family relationships.

#### 3.5.3 Health and Wellbeing

Whether family PA levels had increased or not, most caregivers viewed improved wellbeing as an important outcome. Participants often emphasised improvements in mental wellbeing more so than improvements in PA levels, suggesting this was perceived as the primary advantage. Many caregivers described using PA as a coping strategy: ‘Just as long as I do something a day then I feel better, the kids are better because they've been out’ (P004, F, age 37, 4 children). Physical health effects were also perceived as a key outcome. Losing weight and increasing cardiovascular activity such as swimming were discussed as goals. Some participants described reaching their goals of losing weight and building their strength.

## 4 Discussion

### 4.1 Summary of Findings

Active Families is a community‐based family PA programme delivered by volunteer peer mentors. It was aimed at families from low socio‐economic backgrounds with at least one child who had been referred for early help services. Early help services support children and families as soon as problems emerge and can include help with parenting, wellbeing and communication. Caregiver experiences of family PA were reported to have improved for most during the programme, with positive impacts on relationships, wellbeing and family life. There is evidence of more positive attitudes towards family PA and seeing other family members engaging in PA encouraged family members to be active. Volunteer peer mentors built confidence and provided accountability and encouragement for families to change PA behaviours and increase activity. They provided crucial psychological, emotional and practical support.

Although the coronavirus pandemic impacted delivery, volunteers were able to maintain continuity with their families via digital methods, and some families used PA as a coping mechanism. Some older children did not engage well with the programme and maintaining PA was a challenge for others.

### 4.2 Strengths and Limitations

This study used rigorous qualitative methodology to gain an in‐depth, rich account of participants' views. Telephone interviewing improved convenience and access (Mann and Stewart 2000) for caregivers at home with small children. Participants may have been more likely to discuss sensitive issues compared to face‐to‐face methods (Opdenakker 2006). Reflexive logs were kept during the interview process, allowing the researcher to critically examine their preconceptions (Watt 2007; O'Connor and Joffe 2020). A third of the transcripts were double‐coded by the research team to provide an independent review of coding. The research team were involved in discussing theme iterations, enhancing credibility and confirmability (Carter et al. 2014).

The charity coordinators played a role in initially approaching caregivers. This was appropriate as many families were vulnerable but may have introduced some gatekeeper bias (Oppong 2013). Although data saturation was achieved for the transcripts studied (Saunders et al. 2018), there may have been some perspectives in the population that were not considered. For example, only White British participants were interviewed, which was not fully representative of all families participating in the Active Families programme (88% White British). This study did not explore experiences of the volunteer peer mentors or children. It was performed during significant social restrictions due to the coronavirus pandemic, which impacted the delivery of the Active Families programme.

### 4.3 Comparisons of Study Findings to Literature

#### 4.3.1 Behaviour Change Techniques

Participants described techniques utilised by the volunteer peer mentors (e.g., confidence building) that are represented in literature by the COM‐B model of behaviour (Howlett et al. 2019). This model recognises that behaviour is influenced by many factors, which can be targeted by interventions. The model highlights the mechanisms behind which the volunteer peer mentors enabled families to change their PA behaviours. Caregivers described an increase in their capabilities, with more self‐confidence and physical capacity to engage after interacting with the volunteers. Volunteers introduced activity ideas, increasing opportunity. Regular contact with the volunteer helped to motivate families. All of these aspects were reflected during caregivers' interviews and appeared important for the families partaking in the programme. Therefore techniques that address all aspects of the COM‐B model should be actively considered when delivering Active Families.

#### 4.3.2 Peer Mentoring

However, the volunteers provided much more than assisting with behaviour change. The caregiver–volunteer relationship was vitally important and appeared to fill a gap in parenting and emotional support. This is consistent with evidence from peer volunteer PA programmes for older adults where additional support is a key component (Crozier et al. 2020) and expands this evidence base to include family PA programmes. Caregivers spoke of the adaptations the volunteers made to meet their family's needs, and other literature has shown the importance of tailoring PA programmes to unique family characteristics (Noonan et al. 2017). Providing this holistic, tailored approach may be a key factor in its success. The volunteers adapted delivery during the coronovirus pandemic to include activity packs and phone‐based support. This was well received by participants and led to continued engagement in PA despite a period of adversity, as seen in other family PA programmes delivered virtually during the pandemic (Tripicchio et al. 2023).

#### 4.3.3 Family PA

This study provides some evidence for the hypothesis that if families are active together, they are more likely to motivate each other and maintain PA (Spokes 2021). Children observing their caregiver's involvement in PA was a potential mechanism demonstrated, adding to the existing evidence base on the importance of parental role modelling (Cleland et al. 2011; Timperio et al. 2013; Brown et al. 2016). In contrast, there were a few families where there was minimal child engagement; this tended to be those families with teenage children. This could be explained by other studies in older children where their PA patterns were influenced by friends' support and PA (Springer et al. 2006; Fitzgerald et al. 2012). Friends rather than parents may be more influential at motivating PA behaviour in older children.

### 4.4 Conclusion

The insight from this study provides an understanding of the likely mechanisms behind successful family PA behaviour change and the importance of holistic peer mentor support. This learning can be used locally and nationally to inform family PA interventions, with the aim of improving health and wellbeing throughout the community.

## Author Contributions

**Rebecca Symes:** conceptualisation, methodology, investigation, validation, formal analysis, writing – original draft, writing – review and editing, data curation. **Leah Jayes:** conceptualisation, methodology, investigation, validation, supervision, writing – original draft, writing – review and editing, formal analysis. **Elizabeth Orton:** conceptualisation, methodology, investigation, validation, supervision, writing – original draft, writing – review and editing.

## Ethics Statement

The study was approved by the Faculty of Medicine and Health Sciences (University of Nottingham, UK) Research Ethics Committee (Ref. 22‐0520).

## Consent

Verbal consent was taken from each participant prior to data collection, in line with ethical guidance and approvals outlined from the Faculty of Medicine and Health Sciences (University of Nottingham, UK).

## Conflicts of Interest

The authors declare no conflicts of interest.

## Supporting information


**Data S1.** Supporting Information.

## Data Availability

The data underlying this article cannot be shared publicly to protect the privacy of participants. The data will be shared on reasonable request to the corresponding author.
