# Supplementary material for: Caregiver Experiences of a Peer Mentor Family Physical Activity Programme in England: A Qualitative Interview Study
Source: Child Care Health Dev. 2025 Feb 24;51(2):e70053. doi: 10.1111/cch.70053 (PMC11850952; doi:10.1111/cch.70053)
Supplement: Supplementary file 1 — Data S1. Supporting Information. [file CCH-51-e70053-s001.docx]

|  | **Supplementary Files**  1. Standards for Reporting Qualitative Research (SRQR)  2. Interview guide  **File 1. Standards for Reporting Qualitative Research (SRQR)*** |  |
| --- | --- | --- |
|  | <http://www.equator-network.org/reporting-guidelines/srqr/> |  |
|  |  | **Page/line no(s).** |
| **Title and abstract** | |  |
|  | **Title** - Concise description of the nature and topic of the study Identifying the study as qualitative or indicating the approach (e.g., ethnography, grounded theory) or data collection methods (e.g., interview, focus group) is recommended | P1&2 |
|  | **Abstract** - Summary of key elements of the study using the abstract format of the intended publication; typically includes background, purpose, methods, results, and conclusions | P1 |
|  |  |  |
| **Introduction** | |  |
|  | **Problem formulation** - Description and significance of the problem/phenomenon studied; review of relevant theory and empirical work; problem statement | P2-3 |
|  | **Purpose or research questio**n - Purpose of the study and specific objectives or questions | P3 |
|  |  |  |
| **Methods** | |  |
|  | **Qualitative approach and research paradigm** - Qualitative approach (e.g., ethnography, grounded theory, case study, phenomenology, narrative research) and guiding theory if appropriate; identifying the research paradigm (e.g., post positivist, constructivist/ interpretivist) is also recommended; rationale** | P3 |
|  | **Researcher characteristics and reflexivity** - Researchers’ characteristics that may influence the research, including personal attributes, qualifications/experience, relationship with participants, assumptions, and/or presuppositions; potential or actual interaction between researchers’ characteristics and the research questions, approach, methods, results, and/or transferability | P3-4, 12 |
|  | **Context** - Setting/site and salient contextual factors; rationale** | P3-4 |
|  | **Sampling strategy** - How and why research participants, documents, or events were selected; criteria for deciding when no further sampling was necessary (e.g., sampling saturation); rationale** | P3-4 |
|  | **Ethical issues pertaining to human subjects** - Documentation of approval by an appropriate ethics review board and participant consent, or explanation for lack thereof; other confidentiality and data security issues | Title page |
|  | **Data collection methods** - Types of data collected; details of data collection procedures including (as appropriate) start and stop dates of data collection and analysis, iterative process, triangulation of sources/methods, and modification of procedures in response to evolving study findings; rationale** | P3-4 |
|  | **Data collection instruments and technologies** - Description of instruments (e.g., interview guides, questionnaires) and devices (e.g., audio recorders) used for data collection; if/how the instrument(s) changed over the course of the study | P3-4 |
|  | **Units of study** - Number and relevant characteristics of participants, documents, or events included in the study; level of participation (could be reported in results) | P4,5&6 |
|  | **Data processing** - Methods for processing data prior to and during analysis, including transcription, data entry, data management and security, verification of data integrity, data coding, and anonymization/de-identification of excerpts | P3-4 |
|  | **Data analysis** - Process by which inferences, themes, etc., were identified and developed, including the researchers involved in data analysis; usually references a specific paradigm or approach; rationale** | P3-4 |
|  | **Techniques to enhance trustworthiness** - Techniques to enhance trustworthiness and credibility of data analysis (e.g., member checking, audit trail, triangulation); rationale** | P3-4 |
|  |  |  |
| **Results/findings** | |  |
|  | **Synthesis and interpretation** - Main findings (e.g., interpretations, inferences, and themes); might include development of a theory or model, or integration with prior research or theory | P8-14 |
|  | **Links to empirical data** - Evidence (e.g., quotes, field notes, text excerpts, photographs) to substantiate analytic findings | P7-11 plus Table 2 |
|  |  |  |
| **Discussion** | |  |
|  | **Integration with prior work, implications, transferability, and contribution(s) to the field -** Short summary of main findings; explanation of how findings and conclusions connect to, support, elaborate on, or challenge conclusions of earlier scholarship; discussion of scope of application/generalizability; identification of unique contribution(s) to scholarship in a discipline or field | P12-13 |
|  | **Limitations** - Trustworthiness and limitations of findings | P12 |
|  |  |  |
| **Other** | |  |
|  | **Conflicts of interest** - Potential sources of influence or perceived influence on study conduct and conclusions; how these were managed | Title page & online submission |
|  | **Funding** - Sources of funding and other support; role of funders in data collection, interpretation, and reporting | Title page & online submission |
|  |  |  |
|  | *The authors created the SRQR by searching the literature to identify guidelines, reporting standards, and critical appraisal criteria for qualitative research; reviewing the reference lists of retrieved sources; and contacting experts to gain feedback. The SRQR aims to improve the transparency of all aspects of qualitative research by providing clear standards for reporting qualitative research. |  |
|  |  |  |
|  | **The rationale should briefly discuss the justification for choosing that theory, approach, method, or technique rather than other options available, the assumptions and limitations implicit in those choices, and how those choices influence study conclusions and transferability. As appropriate, the rationale for several items might be discussed together. |  |
|  |  |  |
|  | **Reference:** |  |
|  | O'Brien BC, Harris IB, Beckman TJ, Reed DA, Cook DA. **Standards for reporting qualitative research: a synthesis of recommendations.** *Academic Medicine*, Vol. 89, No. 9 / Sept 2014  **File 2: Interview guide** |  |

NB – Questions 24 and 25 added after pilot interviews.

Introduction

- Thank participant for agreeing to take part in the study.
- Review the purpose of the interview:
  - Interested to hear your experiences of the Active Families programme and doing physical activity as a family.
  - Explain their views will help us understand how people engage in physical activity as a family and develop similar programmes in the future.
- Explain right to withdraw consent
- ‘The information you tell us in these interviews will be kept confidential. However, the exception to this would be if you share anything that I consider would put you or a member of your family at risk.  If this happens I will let you know that I need to pass this information on in order for you to get the support you need.’
- Reaffirm that the interviewee is happy for the interview to audio-recorded and anonymised quotes could be used in future reports or publications.
- Check consent form and if participant still happy to proceed with interview.
- Ask participant if they have any questions before starting the interviews.

Background

*In the first section I am going to ask you about your family set up and any activities you were doing before you got involved with Active Families.*

1. Which area of Leicestershire do your family live in?
2. Can you tell me about the family members you are living with at the moment?
3. Before you started Active Families, can you tell me a bit about any physical activity you and your family were doing?
   - *Probes:*
   - How often?
   - What activities?
   - Who was involved in these activities?
   - Did you do any physical activity as a family?
   - Was anyone supporting you with these activities?
4. How did you and your family feel about doing physical activity at this time?
   - *Probes:*
   - What motivated you to do physical activity?
   - How did physical activity make you feel?

Initial involvement with Active Families

*In the next section I am going to ask you about that initial contact with Active Families and why you got involved.*

1. How did you and your family first hear about and get involved with Active Families?
2. Why did you and your family get involved with Active Families?
   - *Probes:*
   - Was there anything you wanted to achieve by being involved?
3. What did you understand was the aim of Active Families?
   - *Probes:*
   - Were you provided with any background as to why Active Families was being run?

During the Active Families Programme

*In the next section I am going to ask you about your time involved with the Active Families programme.*

1. How long has your family been involved with Active Families?
   - *Probes:*
   - If you have finished Active Families, how long were you involved with the programme for?
   - If you finished the Active Families programme early, why was this?
2. Who in your family has been involved with Active Families?
3. Can you tell me about the physical activity your family has/had been doing during the Active Families programme?
   - *Probes:*
   - How often?
   - What activities?
   - Who is/was involved in these activities?
   - Do/did you do any physical activity as a family? Why/why not?
   - Who has/had been supporting you with these activities?
   - Do/did you do these activities with the volunteers? Or on your own?
4. How do/did you and your family feel about doing physical activity now/then?
   - *Probes:*
   - What motivates/d you to do physical activity?
   - How does/did the physical activity make you feel?
5. Has Active Families changed the amount of physical activity you are doing as a family?
   - *Probes:*
   - How has this changed?
   - Amount of physical activity?
   - Types of physical activity?
   - Which family members have been involved?
   - Why has it changed?
6. Tell me about your experience of doing physical activity as a family?
   - *Probes:*
   - What are the good things about doing physical activity as a family?
   - Are there any difficulties?
7. During the Active Families programme, what kinds of things affect/affected how much physical activity you are/were doing as a family?
   - *Probes:*
   - Are there things that help/helped you to do more physical activity?
   - Are there things that make/made it more difficult or stop/stopped you doing physical activity? Why?
8. Tell me about your experiences of working with [enter volunteer name]?
   - *Probes:*
   - How often have you been in contact?
   - What do they help with?
   - Is there anything your have found particularly helpful?
   - Is there anything you have not found so helpful?
9. How have the activities you and your family have been doing changed during your time working with [enter volunteer name]?
   - *Probes:*
   - Could you give me some examples?
   - Why have these activities changed?
10. We have had a very challenging time with restrictions due to coronavirus, how has this affected physical activity in your family?
    - *Probes:*
    - Have your physical activity levels changed? Why?
    - Has it made physical activity more difficult? Why?
    - Has it made physical activity easier? Why?
    - Have the activities you have been able to do changed?
    - How have you adapted as a family?
    - How has physical activity made you and your family feel?
11. Tell me how [enter volunteer name] has supported you during this challenging time?

Physical activity after Active Families (optional for families who have finished/stopped programme)

*In the next section we will talk about physical activity after Active Families.*

1. Can you tell me a bit about the physical activity you and your family are doing now you have finished the Active Families programme?
   - *Probes:*
   - How often?
   - What activities?
   - Who is involved in these activities?
   - Have the activities you have been doing changed?
   - Do you do any physical activity as a family? Why/why not?
   - Who has been supporting you with these activities?
2. How do you and your family feel about doing physical activity now?
   - What motivates you to do physical activity?
   - How does the physical activity make you feel?
3. Now you have finished the Active Families programme, what kinds of things affect how much physical activity you are doing as a family?
   - *Probes:*
   - Are there things that help you to do more physical activity?
   - Are there things that make it more difficult or stop you doing physical activity? Why?

Reflections on involvement in Active Families

*In the final section we are going to discuss your overall reflections of Active Families.*

1. Earlier on you explained you got involved with Active Families because [enter reason given from question 6]. Do you feel that Active Families has helped you with this?
   - *Probes:*
   - Why? Why not?
2. What is the best thing that has come out of being involved with Active Families for you and your family?
3. How do you see physical activity being part of you and your family’s routine in the future?
4. Do you or your family have any physical activity goals for the future?
5. Active Families focuses on supporting families to become more physically active but have you also seen any wider benefits?
   - *Probes:*
   - Any changes to relationships?
   - Any changes to home life?
   - Any changes to family life?
   - Any changes to wellbeing or health?
6. Thinking about if Active Families was introduced to other areas of England, is there anything you would change about Active Families?

Conclusion

- Is there anything we haven’t talked about today relating to Active Families that you would like to add?
- Are there any questions you would like to ask me?
- Explain to participant that they have reached the end of the interview.
- Again, remind the participant about confidentiality.
- Thank participant for their time.
- Discuss inconvenience allowance (voucher) and how this will be organised.
